# Modelling concentrations of antimicrobial drugs: comparative pharmacokinetics of cephalosporin antimicrobials and accuracy of allometric scaling in food-producing and companion animals

**DOI:** 10.1186/s12917-016-0817-2

**Published:** 2016-09-06

**Authors:** Femke J. Taverne, Ingeborg M. van Geijlswijk, Dick J. J. Heederik, Jaap A. Wagenaar, Johan W. Mouton

**Affiliations:** 1 The Netherlands Veterinary Medicines Authority (SDa), Yalelaan 114, 3584 CM Utrecht, The Netherlands; 2 Institute for Risk Assessment Sciences, Utrecht University, Yalelaan 2, 3584 CM Utrecht, The Netherlands; 3 Pharmacy Department, Faculty of Veterinary Medicine, Utrecht University, Yalelaan 106, 3584 CM Utrecht, The Netherlands; 4 Department of Infectious Diseases & Immunology, Faculty of Veterinary Medicine, Utrecht University, Yalelaan 1, 3584 CL Utrecht, The Netherlands; 5 Central Veterinary Institute, Wageningen UR, Houtribweg 39, 8221 RA Lelystad, The Netherlands; 6 Department of Medical Microbiology and Infectious Diseases, Erasmus MC, Wytemaweg 80, 3015 CN Rotterdam, The Netherlands

**Keywords:** Mathematical models, Allometric scaling, Pharmacokinetics, Cephalosporins, Antimicrobials, Food-producing animals, Companion animals

## Abstract

**Background:**

To optimize antimicrobial dosing in different animal species, pharmacokinetic information is necessary. Due to the plethora of cephalosporin antimicrobials and animal species in which they are used, assessment of pharmacokinetics in all species is unfeasible. In this study we aimed to describe pharmacokinetic data of cephalosporins by reviewing the available literature for food producing and companion animal species. We assessed the accuracy of interspecies extrapolation using allometric scaling techniques to determine pharmacokinetic characteristics of cephalosporins in animal species for which literature data is unavailable. We assessed the accuracy of allometric scaling by comparing the predicted and the published pharmacokinetic value in an animal species/humans not included in the allometric modelling.

**Results:**

In general, excretion of cephalosporins takes place mainly through renal mechanisms in the unchanged form and volume of distribution is limited in all animal species. Differences in plasma protein binding capacity and elimination half-life are observed but available information was limited. Using allometric scaling, correlations between body weight (BW) and volume of distribution (Vd) and clearance (Cl) were *R* ^*2*^ > 0.97 and *R* ^*2*^ > 0.95 respectively for ceftazidime, ceftiofur, cefquinome and cefepime but not ceftriaxone. The allometric exponent ranged from 0.80 to 1.31 for Vd and 0.83 to 1.24 for Cl. Correlations on half-life ranged from R^2^ 0.07–0.655 (literature) and R^2^ 0.102–0.876 (calculated).

**Conclusions:**

Allometric scaling can be applied for interspecies extrapolation of cephalosporin pharmacokinetic parameters Vd and Cl, but not elimination half-life. We hypothesize that the accuracy could be improved by using more refined scaling techniques.

**Electronic supplementary material:**

The online version of this article (doi:10.1186/s12917-016-0817-2) contains supplementary material, which is available to authorized users.

## Background

Antimicrobials are used in both food-producing animals like cattle, pigs, poultry and rabbits and companion animals such as dogs, cats and horses. These animals are known to be potential reservoirs of microorganisms carrying antimicrobial resistance genes [1–5]. Emergence of resistance to antimicrobials in pathogens such as *Enterobacteriaceae, Staphylococcus spp.* and *Streptococcus spp.* has led to an increased awareness of the need to optimize dosing regimens [6, 7]. Third and fourth generation cephalosporins mainly target Gram-negative microorganisms such as *Enterobacteriaceae (*including *Escherichia coli*) and have decreased activity against Gram-positive microorganisms such as *Staphylococcus spp.* and *Streptococcus spp.*, the primary target of earlier generation cephalosporins. However, before dosage regimens can be optimized detailed knowledge of the pharmacokinetics of cephalosporins is needed. We hypothesize that these data are available for major food-producing animal species such as cattle and pigs, but less so for species like rabbits and companion animals.

The objective of this study is to gain accurate pharmacokinetic data of cephalosporins in animal species for which these data are not available from literature. We intend to calculate these missing pharmacokinetic data by interspecies extrapolation of known pharmacokinetic data in other animal species using allometric scaling. The allometric scaling technique is one of the techniques used to determine the first-in-human dose of new drugs for clinical trials, which is also extrapolation of pharmacokinetic data between animal species [8, 9]. Studies have already proven allometric scaling can be applied for extrapolation of pharmacokinetic data of cephalosporins, but these studies were based on data from a limited selection of animal species [10, 11]. In this study we collect available pharmacokinetic data on cephalosporins of a range of food-producing (cattle, pigs, chickens, rabbits, horses) and companion animal (dogs, cats, rabbits, horses) species and assess the accuracy of interspecies extrapolation by allometric scaling of pharmacokinetic data over this broad range of animal species. We have opted for allometric scaling because this technique is less time consuming and requires less input parameters than more refined methods for interspecies extrapolation, like physiologically based pharmacokinetic (PBPK) modelling [12–14]. Also, allometric scaling is a more widely applied and understood technique.

In this study we present and review the pharmacokinetic data of cephalosporins available from literature for a wide range of animal species. Furthermore we subject basic pharmacokinetic parameters (clearance (Cl), volume of distribution (Vd) and elimination half-life (t½)) to allometric analysis and assess the accuracy of this method based on a larger subset of animal species than what is usually applied in allometric analysis.

## Methods

### Data collection

A search for literature reporting pharmacokinetic data of cephalosporins authorized for veterinary use and of cephalosporins authorized for human use and which are known to be used off-label in companion animals was performed in PubMed, Scopus and Google Scholar. Search statements included combinations of the following terms: pharmacokinetics, veterinary, cephalosporins and names of several specific animal species and/or cephalosporins. No language restriction was applied in the search. References describing pharmacokinetics of combination therapies were excluded from the results as other compounds can potentially interfere with the pharmacokinetics of the cephalosporin(s) studied. Data retrieved after administration by other routes than the intravenous route was excluded in order to eliminate the influence of absorption pharmacokinetics. Studies in that were performed with experimental (non-therapeutic) dosages were also excluded.

### Allometric analysis

Allometric scaling based on body weight was applied to the collected pharmacokinetic data, to the parameters Vd, Cl and t½. These pharmacokinetic parameters are the core parameters reported in pharmacokinetic studies and therefore for these parameters most data is available. Data on other pharmacokinetic indices is limited, making allometric analysis unfeasible. The availability of data and importance in the description of pharmacokinetic behaviour of a compound makes Vd, Cl and t½ the best candidates for allometric scaling.

For the analysis the allometric equation (Eq. 1) was used, which can be written as follows [8, 9, 11, 15]:

1 $$ Y=a{W}^b $$

where *Y* is the pharmacokinetic parameter of interest, *W* is the body weight in kg, *a* is the coefficient of the allometric equation and *b* is the allometric exponent. Log-transformed this equation turns into a linear function and the equation (Eq. 2) is represented as follows [8, 11]:

2 $$ \log Y= \log a+b \log W $$

with log *a* being the intercept and *b* being the slope. As *Y* and *W* were known parameters, values for *a* and *b* could be calculated with a trend line. The trend line also enabled assessment of the correlation between pharmacokinetic values for different animal species.

Allometric scaling was performed for those active substances with pharmacokinetic data obtained after intravenous administration available for at least four different species of animals to allow for robust allometric analysis. Young animals were considered an extra animal species due to often substantial differences in body weight and potential differences in pharmacokinetics compared to adults. The mean body weight and values for Vd, Cl and t½ were all retrieved from the collected pharmacokinetic studies and no other sources were used.

To evaluate the accuracy of extrapolation of pharmacokinetics across animal species with allometric models, pharmacokinetic parameters in humans and other animal species were extrapolated (based on the reported mean animal body weight) and then compared with the observed pharmacokinetic parameters for the species. Ceftazidime, authorized for human use, was evaluated with and without human pharmacokinetic data. Ceftiofur and cefquinome pharmacokinetic data from other animal species were used as reference due to the exclusively veterinary use of these cephalosporins.

Analyses were performed using RStudio Version 0.98.490. 2013 (RStudio inc. Boston, USA) and Excel 2010 (Microsoft, Redmond, Washington, USA).

## Results

### Pharmacokinetics of cephalosporins

The collected pharmacokinetic data on cephalosporins are presented in Additional file 1: Table S1. Pharmacokinetics of 1st and 2nd generation cephalosporins for the included animal species were available from 15 studies (horses *n* = 7, dogs *n* = 4, cats *n* = 2 and cattle *n* = 2) involving 5 different cephalosporins (cefadroxil, cefazolin, cefapirin, cefalexin and cefoxitin). An interspecies difference was observed for plasma protein binding between horses and cattle for cefazolin (75 % in cattle, compared to 8.3 % in horses). Comparing different cephalosporins over all species, cefazolin had the shortest elimination half-life (ranging from 0.62 h in cattle to 1.23 h in dogs) and cefalexin the longest (1.38 h in dogs to 2.02 h in horses). Volume of distribution was limited for all cephalosporins (ranging from 0.135 L/kg (cefazolin in horses) to 0.374 L/kg (cefadroxil in horses)). Clearance ranged from 0.140 L/kg/h (cefalexin and cefoxitin in cats) to 0.598 L/kg/h (cefapirin in horses). Data was too limited for comparison of different cephalosporins within each animal species, except in horses. No substantial differences exist between pharmacokinetics of different cephalosporins in horses; t½ is short (ranging from 0.63 to 2.02 h), volume of distribution is limited (0.135–0.374 L/kg) and excretion is mainly through renal mechanisms for all compounds with a clearance of 0.204–0.598 L/kg/h.

For 3rd and 4th generation cephalosporins 38 studies (cattle *n* = 9, chickens *n* = 2, pigs *n* = 3, horses *n* = 10, dogs *n* = 9, cats *n* = 4 and rabbits *n* = 4) met the inclusion criteria. Some studies covered multiple animal species. Clinically relevant interspecies differences in elimination half-life were observed for ceftiofur, half-life ranged from 4.23 h in chicken to 21.5 h in horses. Intermediate half-lives were found for calves (16.1 h), pigs (11.01 h) and foals (5.17–8.08 h). In general, elimination half-life was short for the other cephalosporins (except cefovecin) with limited interspecies differences: cefoperazone 0.50–2.13 h, ceftazidime 0.73–2.31 h, ceftriaxone 0.81–3.25 h, cefotaxime 0.60–3.48 h, cefquinome 0.85–2.77 h, cefepime 1.09–2.38 h and cefpirome 0.79–1.48 h.

Excretion of 3rd and 4th generation cephalosporins is mainly renal and unchanged. Two 3rd and 4th generation cephalosporins are not excreted unchanged. These are ceftiofur, which is metabolized by the liver to active desfuroylceftiofur and cefotaxime which is metabolized to active desacetylcefotaxime (see Additional file 1: Table S1). Depending on the cephalosporin, elimination can be through glomerular filtration with or without the addition of tubular secretion. For ceftazidime it was reported for cats [16] that glomerular filtration is the mechanism of excretion. This is confirmed by comparing the clearance for ceftazidime in cats (0.190 L/kg/h) to the glomerular filtration rate (GFR) measured in cats (renal inulin clearance) of 0.182 L/kg/h) [17]. For ceftriaxone it is reported that the mechanism of excretion in cats is through glomerular filtration and tubular secretion and/or non-renal excretion with a clearance of 0.370 L/kg/h [18], which exceeds the GFR in cats. In dogs the measured GFR (renal inulin clearance) is 0.235 L/kg/h [17]. The clearance of ceftazidime in dogs is reported to be 0.192 L/kg/h [19] and 0.228 L/kg/h [20]. This relates very well to the GFR in dogs. The reported clearance of ceftriaxone is 0.217 L/kg/h [21] in dogs, which is also close to the GFR. This might indicate that ceftazidime is excreted exclusively through glomerular filtration in both dogs and cats, yet, for ceftriaxone this only seems to be the case for dogs and not for cats. The renal clearance of cefquinome ranges from 0.191 to 0.221 L/kg/h [22] in dogs. Although the mechanism of excretion is not mentioned in the study, it correlates so well to the GFR in dogs that also cefquinome is probably excreted exclusively through glomerular filtration in dogs. Data on cats is not available.

### Allometric analysis

**Fig. 1:**
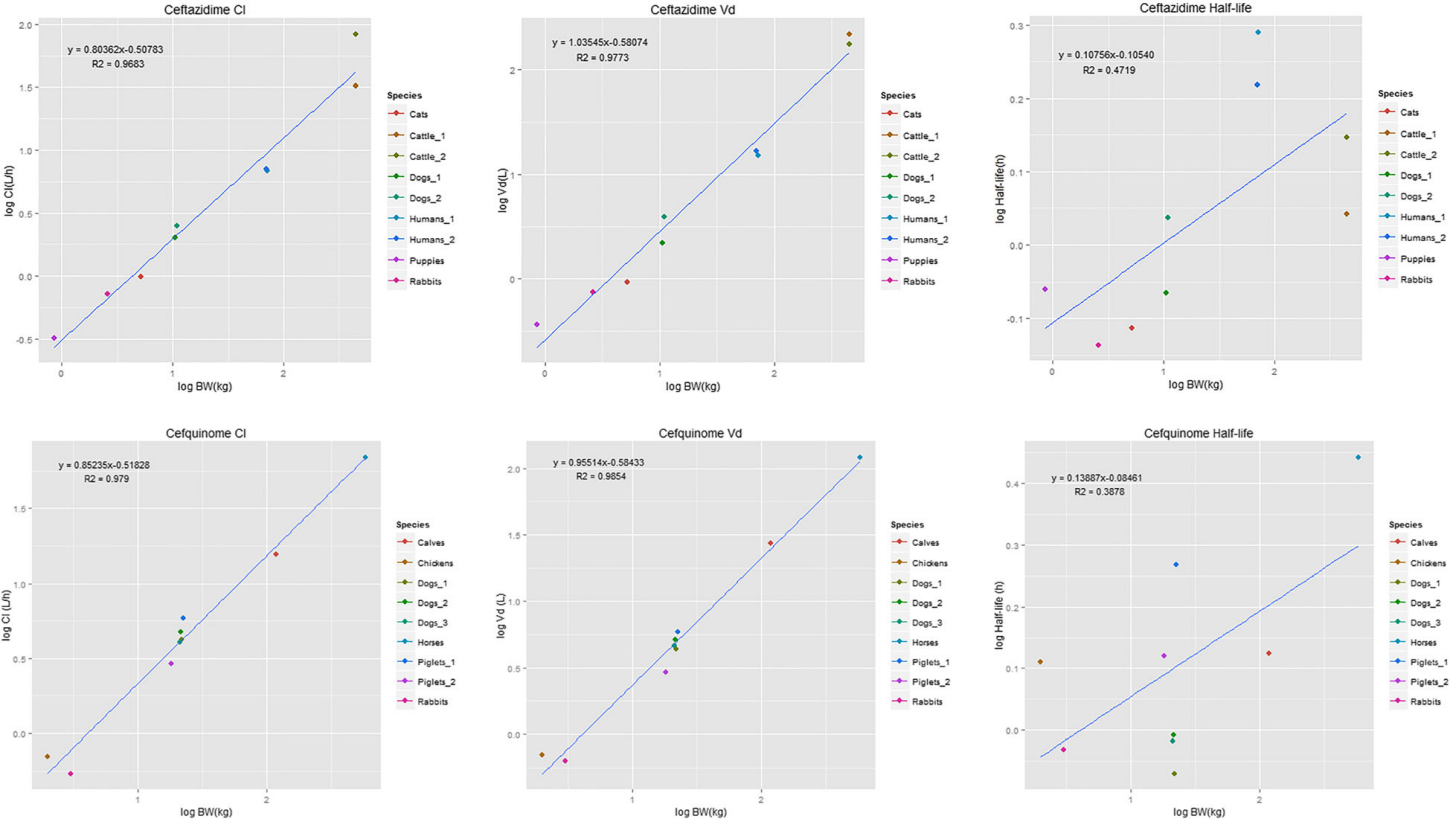
Two examples of allometric analysis performed on pharmacokinetic data of cephalosporins. Figure shows clearance, volume of distirubution and half-life of ceftazidime (3rd generation cephalosporin) and cefquinome (4th generation cephalosporin). Ceftazidime is also used in human medicine, human data are included in this figure and the equation and R^2^ shown are based on allometric analysis including human data

Sufficient pharmacokinetic data to apply allometric analysis was available for five cephalosporins, cefquinome, ceftriaxone, ceftazidime, ceftiofur and cefepime. The results of the allometric scaling regression analysis (allometric coefficient, allometric exponent and correlation (R^2^)) of volume of distribution, clearance and elimination half-life are shown in Table 1. Graphs on the allometric scaling of ceftazidime (including human data) and cefquinome (exclusively veterinary use) are presented in Fig. 1. The allometric analyses of cefepime, ceftriaxone and ceftiofur are shown in Additional file 1: Figure S1 t/m S3. Additional file 1: Figure S4 shows the allometric analysis for ceftazidime excluding human data. For ceftazidime, ceftiofur, cefquinome and cefepime (but not ceftriaxone) correlations between body weight and both parameters volume of distribution and clearance were high (*R* ^*2*^ > 0.97 and *R* ^*2*^ > 0.95 respectively). The allometric exponent for all five cephalosporins ranged from 0.80 to 1.31 for Vd and 0.83 to 1.24 for Cl. Half-life proved to be less predictable using allometric scaling with R^2^ 0.067–0.655 based on the values for half-life retrieved from literature. Calculating half-life (t½ = (ln2*Vd)/Cl) improved correlation to a range of R^2^ 0.102–0.876. The calculated half-life per study is available in Additional file 1: Table S1. For cefepime correlation improved the most after calculating (from R^2^ 0.628 to 0.876). Correlations for ceftriaxone and ceftazidime remained almost equal (R^2^ 0.067 versus 0.102 and R^2^ 0.655 versus 0.662 respectively) and dropped for ceftiofur and cefquinome (R^2^ 0.481 versus 0.128 for ceftiofur and R^2^ 0.388 versus 0.243 for cefquinome).

**Table 1:** Allometric scaling of pharmacokinetics of different cephalosporins in animals

Cephalosporin	Included animal species	Total no. of animals included (*n)*	Pharmacokinetic parameter	Allometric coefficient	Allometric exponent	R^2^ (R^2^ including human data)	R^2^ with calculated t½	References
Ceftriaxone	Dogs, cats, foals, horses	22	Half-life	1.9820	0.0896	0.0672	0.102	[18, 21, 31, 32]
			Volume of distribution	0.9213	0.8044	0.7173		
			Clearance	0.4569	0.8854	0.9158		
Ceftazidime	Dogs (puppies and adults), cats, cattle, rabbits	34	Half-life	0.7690	0.0768	0.6550 (0.4719)	0.662	Animal + human data [16, 19, 20, 33–35]:
			Volume of distribution	0.2670	1.0611	0.9829 (0.9773)		
			Clearance	0.3158	0.8306	0.9787 (0.9683)		
Ceftiofur	Calves, chickens, foals, horses	68	Half-life	3.7440	0.2155	0.4811	0.128	[36–41]
			Volume of distribution	0.1485	1.3129	0.9875		
			Clearance	0.0210	1.2431	0.9552		
Cefquinome	Dogs, calves, piglets, chickens, horses, rabbits	51	Half-life	0.8230	0.1389	0.3878	0.243	[22, 42–45]
			Volume of distribution	0.2604	0.9551	0.9854		
			Clearance	0.3032	0.8524	0.9790		
Cefepime	Dogs, calves, foals, horses	29	Half-life	0.7337	0.1787	0.6284	0.876	[46–49]
			Volume of distribution	0.1174	1.1347	0.9783		
			Clearance	0.1827	0.8439	0.9725		

For details on pharmacokinetic data, see Additional file 1: Table S1. In the presented results both young and adult animals were included in the allometric analysis. The values reported here are excluding any human data, except for ceftazidime, where allometric analysis was performed both with and without human data

To further assess accuracy of extrapolation of pharmacokinetics to humans for ceftriaxone, ceftazidime and cefepime, two human pharmacokinetic studies per cephalosporin were used to compare extrapolated pharmacokinetics to observed data. For ceftazidime allometric scaling was repeated with the human data included, to assess the changes to the allometric equation and correlation coefficient. The scaling including human data is shown in Fig. 1 (without human data in Additional file 1: Figure S4). As can be seen in the figures and Table 1 the correlation drops for all three pharmacokinetic parameters, especially for the elimination half-life (from R^2^ 0.655 to 0.472). For ceftriaxone and cefepime no allometric scaling including human data was performed. All studies were performed on healthy volunteers, as pharmacokinetics used for the allometric model were also mostly assessed in healthy animals. For ceftiofur three additional animal species were used with a wide range of body weights, namely goats, camels and Asian elephants. For cefquinome studies in ducks and buffalo were used. Data and results are presented in Table 2. Pharmacokinetics for cefepime could be extrapolated to humans with the animal based allometric equation. For ceftazidime the model overestimated the pharmacokinetic values. Observed Vd was 30–40 % lower than the predicted value and observed Cl was 35–40 % lower than predicted. For ceftriaxone the model overestimated the observed value for Vd about 4 times (extrapolated 0.4 L/kg, observed 0.1 L/kg) and the value for Cl about 23 times (extrapolated 0.280 L/kg/h, observed 0.012 L/kg/h). Allometric scaling of ceftiofur was only accurate for clearance in goats. Pharmacokinetic values for cefquinome could be extrapolated to ducks with reasonable accuracy, but the clearance in buffalo was overestimated (extrapolated 0.149 L/kg/h, observed 0.061 L/kg/h).

**Table 2:** Extrapolated volume of distribution and clearance by allometric scaling versus volume of distribution and clearance

Cephalosporin	(Animal) species	Reported body weight (kg)	Extrapolated volume of distribution (L/kg)	Extrapolated clearance (L/kg/h)	Observed volume of distribution (L/kg)	Observed clearance (L/kg/h)	Reference
Ceftriaxone	Human	72	0.40	0.28	0.11 ± 0.02	0.012 ^*a*^	Paradis [34]
	Human	79	0.39	0.28	0.12 ± 0.02	0.011 ^*c*^	Chiu [50]
Ceftazidime	Human	72.0	0.35	0.15	0.21 ± 0.03	0.095 ± 0.014 ^*a*^	Paradis [34]
	Human	70.8	0.35	0.15	0.204 ± 0.02	0.069 ± 0.011^*e*^	Paulfeuerborn [35]
Cefepime	Human	70	0.21	0.09	0.23	0.092 ^*b*^	Bacher [51]
	Human	74	0.21	0.09	0.25	0.101 ^*b*^	Barbhaiya [52]
Ceftiofur	Goat (non-lactating)	58.5	0.53	0.06	0.25	0.067 ^*a*^	Courtin [53]
	Camel	400	0.97	0.09	0.13 ± 0.03	0.03 ± 0.001	Goudah [54]
	Asian elephant	3530	1.91	0.15	0.51 ± 0.29	0.069 ± 0.043	Dumonceaux [55]
Cefquinome	Duck	2.2	0.25	0.27	0.41 ± 0.04	0.22 ± 0.02	Yuan [56]
	Buffalo	123	0.21	0.15	0.26 ± 0.006	0.061 ^*d*^	Dinakaran [57]

Data are observed values from pharmacokinetic studies after intravenous administration of cephalosporin to healthy subjects. Values are reported as value ± SD where possible

^a^ Recalculated from ml/kg/min. ^b^ Recalculated from ml/min. ^c^ Recalculated from L/h. ^d^ Recalculated from ml/kg/h. ^e^ Recalculated from ml/min/1.73 m^2^ (assuming 1.73 m^2^ equals a 70 kg weighing human)

## Discussion

Pharmacokinetic data on cephalosporins in different animal species presented here shows that, independent of animal species, cephalosporins have a limited distribution in body tissues other than plasma, undergo no or little biotransformation and the primary route of elimination is through renal mechanisms with a generally short elimination half-life. Our study underlines the possibility of interspecies extrapolation of pharmacokinetic parameters of cephalosporins with allometric scaling, at least for volume of distribution and clearance and less for elimination half-life. In other studies, allometric scaling of a variety of drugs (not cephalosporins) also showed good overall correlation of volume of distribution and clearance with body weight across species, especially when at least one large animal (for example cattle or horses) were added to the scaling besides laboratory animals such as mice, rats and dogs [8]. Only one of the four cephalosporins used in allometric analyses by Riviere et al. [11] showed a high correlation between body weight and elimination half-life (R^2^ of 0.97 for cefapirin). For the other three cephalosporins correlation was poor (R^2^ 0.03 for cefamandole, 0.07 for cefazolin and 0.20 for cefalothin). These findings are in agreement with the results presented in this paper. It should be noted, however, that for some drugs elimination half-life does scale well with body weight, as shown with carboplatin in several avian species [23] and for example tetracycline (R^2^ 0.97) by Riviere et al. [11] and should therefore still be considered as a scaling parameter when performing allometric analysis.

Although the pharmacokinetic profile of cephalosporins in general was comparable between animal species some specific differences were noted. One of the observed differences was in the plasma protein binding. Values for plasma protein binding were only scarcely reported in the reviewed studies and therefore impossible to extrapolate to other animal species. Results in laboratory animals showed high protein binding for ceftriaxone in rabbits and rodents [24] and an overall higher plasma protein binding in rabbits compared to rodents and dogs, independent of cephalosporin [25]. The effect of plasma protein binding on pharmacokinetics can be substantial for drugs with high protein binding and renal clearance, as is reported for cefovecin. High plasma protein binding is thought to account for the extremely long elimination half-life of cefovecin in cats and dogs [26, 27]. but in order to understand the exact impact of plasma protein binding on pharmacokinetics of cephalosporins more experimental data is needed.

Also, some cephalosporins like ceftriaxone, are eliminated through the faeces as well as through urine, but not to the same extent in all animal species. Pharmacokinetics of drugs that are primarily excreted biliary prove harder to extrapolate due to differences in biliary excretion and bile flow rates between animal species. Dogs and chickens are good biliary excretors, while cats are moderate and rabbits and humans are poor biliary excretors [28]. Furthermore, interspecies differences in enterohepatic circulation and urine pH exist that can influence elimination pharmacokinetics [11]. Carnivores such as dogs and cats generally have acidic urine (pH 5.5–7.0) while herbivores like cattle and horses have alkaline urine (pH 7.0–9.0) [28]. Extrapolation can also be expected to be less accurate for active compounds undergoing capacity-limited hepatic biotransformation rather than flow limited hepatic biotransformation and excretion as interspecies differences exist for these drug metabolism processes. This may contribute to the differences in metabolism of ceftiofur, which probably leads to the poor extrapolation of pharmacokinetics across animal species observed here. Cats, for example, are known to be poor in glucuronidation while dogs on the other hand are deficient acetylators and pigs lack sulfation capacity [28]. Cattle is known to metabolize ceftiofur very efficiently to desfuroylceftiofur [29], perhaps to a greater extent than other animal species. It should also be noted that the young age of the calves could have influenced the results, as young animals have relatively more water and less fat than adult animals (influencing volume of distribution) and organs involved in drug metabolism and elimination still mature in the first few months of life [28].

Finally, it could be hypothesized that coprophagy (or caecotrophy) increases gut exposure to antimicrobials and alters pharmacokinetics of active substances in animals that display this behaviour (such as rabbits, who eat the soft parts of their own excrement, but also pigs, horses and dogs who eat the excrements from other animals [30]). However, no literature is available to assess the significance on these processes and in our results we observed no particular differences in pharmacokinetics in rabbits compared to other animal species.

Allometric scaling of the pharmacokinetics of cephalosporins will assist the parametrization of models for simulation of drug distribution in food-producing and companion animals, such as PBPK models. Where allometric scaling is applied for extrapolation of pharmacokinetic values, PBPK models can extrapolate plasma and tissue concentration-time curves of chemical compounds across animal species, which is ideal for dose optimization of antimicrobials for different animal species.

## Conclusion

Pharmacokinetic behaviour of cephalosporin antimicrobials is in general very similar between animal species. It was shown that extrapolation of pharmacokinetic values for volume of distribution and clearance of most cephalosporins across food-producing and companion animal species can be performed using allometric scaling.

## Additional file

Additional file 1: Pharmacokinetics of cephalosporins in food producing and companion animal species obtained from literature and presented in a table with literature references. We also provide additional graphs displaying the allometric analysis of cefepime, ceftriaxone, ceftiofur and ceftazidime. (PDF 666 kb)

## Abbreviations

Cl: Clearance of a compound from the body
R^2^: Correlation
t½: Elimination half-life of the compound
Vd: Volume of distribution of a compound in the body

## References

[1] Hordijk J, Wagenaar JA, van de Giessen A, Dierikx C, van Essen-Zandbergen A, Veldman K, Kant A, Mevius D (2013). Increasing prevalence and diversity of ESBL/AmpC-type beta-lactamase genes in Escherichia coli isolated from veal calves from 1997 to 2010. J Antimicrob Chemother.

[2] Hordijk J, Schoormans A, Kwakernaak M, Duim B, Broens E, Dierikx C, Mevius D, Wagenaar JA (2013). High prevalence of fecal carriage of extended spectrum beta-lactamase/AmpC-producing Enterobacteriaceae in cats and dogs. Front Microbiol.

[3] Snow LC, Warner RG, Cheney T, Wearing H, Stokes M, Harris K, Teale CJ, Coldham NG (2012). Risk factors associated with extended spectrum beta-lactamase Escherichia coli (CTX-M) on dairy farms in North West England and North Wales. Prev Vet Med.

[4] Cavaco LM, Abatih E, Aarestrup FM, Guardabassi L (2008). Selection and persistence of CTX-M-producing Escherichia coli in the intestinal flora of pigs treated with amoxicillin, ceftiofur, or cefquinome. Antimicrob Agents Chemother.

[5] Dierikx C, van der Goot J, Fabri T, van Essen-Zandbergen A, Smith H, Mevius D (2013). Extended-spectrum-beta-lactamase- and AmpC-beta-lactamase-producing Escherichia coli in Dutch broilers and broiler farmers. J Antimicrob Chemother.

[6] Kouyos RD, Metcalf CJ, Birger R, Klein EY, Abel zur Wiesch P, Ankomah P, Arinaminpathy N, Bogich TL, Bonhoeffer S, Brower C, Chi-Johnston G, Cohen T, Day T, Greenhouse B, Huijben S, Metlay J, Mideo N, Pollitt LC, Read AF, Smith DL, Standley C, Wale N, Grenfell B (2014). The path of least resistance: aggressive or moderate treatment?. Proc Biol Sc.

[7] Goessens WHF (2007). Role of ceftazidime dose regimen on the selection of resistant Enterobacter cloacae in the intestinal flora of rats treated for an experimental pulmonary infection. J Antimicrob Chemother.

[8] Mahmood I (2007). Application of allometric principles for the prediction of pharmacokinetics in human and veterinary drug development. Adv Drug Deliv Rev.

[9] Hunter RP. Interspecies allometric scaling. Comparative Veterinary Pharmacology. Handb Exp Pharmacol. Springer. 2010;199(199):139–57. 10.1007/978-3-642-10324-7_6 20204586

[10] Richter WF, Heizmann P, Meyer J, Starke V, Lave T (1998). Animal pharmacokinetics and interspecies scaling of Ro 25–6833 and related (lactamylvinyl)cephalosporins. J Pharm Sci.

[11] Riviere JE, Martin-Jimenez T, Sundlof SF, Craigmill AL (1997). Interspecies allometric analysis of the comparative pharmacokinetics of 44 drugs across veterinary and laboratory animal species. J Vet Pharmacol Ther.

[12] Lin Z, Li M, Gehring R, Riviere JE (2015). Development and application of a multiroute physiologically based pharmacokinetic model for oxytetracycline in dogs and humans. J Pharm Sci.

[13] Yuan LG, Luo XY, Zhu LX, Wang R, Liu YH (2011). A physiologically based pharmacokinetic model for valnemulin in rats and extrapolation to pigs. J Vet Pharmacol Ther.

[14] Thiel C, Schneckener S, Krauss M, Ghallab A, Hofmann U, Kanacher T, Zellmer S, Gebhardt R, Hengstler JG, Kuepfer L (2015). A systematic evaluation of the use of physiologically based pharmacokinetic modeling for cross-species extrapolation. J Pharm Sci.

[15] West GB, Brown JH (2005). The origin of allometric scaling laws in biology from genomes to ecosystems: towards a quantitative unifying theory of biological structure and organization. J Exp Biol.

[16] Albarellos GA, Ambros LA, Landoni MF (2008). Pharmacokinetics of ceftazidime after intravenous and intramuscular administration to domestic cats. Vet J.

[17] Von Hendy-Willson VE, Pressler BM (2011). An overview of glomerular filtration rate testing in dogs and cats. Vet J.

[18] Albarellos GA, Kreil VE, Landoni MF (2007). Pharmacokinetics of ceftriaxone after intravenous, intramuscular and subcutaneous administration to domestic cats. J Vet Pharmacol Ther.

[19] Kita Y, Yamazaki T, Imada A (1992). Comparative pharmacokinetics of SCE-2787 and related antibiotics in experimental animals. Antimicrob Agents Chemother.

[20] Sakamoto H, Hatano K, Higashi Y, Mine Y, Nakamoto S, Tawara S, Kamimura T, Matsumoto F, Kuwahara S (1993). Animal pharmacokinetics of FK037, a novel parenteral broad-spectrum cephalosporin. J Antibiot (Tokyo).

[21] Rebuelto M, Albarellos G, Ambros L, Kreil V, Montoya L, Bonafine R, Otero P, Hallu R (2002). Pharmacokinetics of ceftriaxone administered by the intravenous, intramuscular or subcutaneous routes to dogs. J Vet Pharmacol Ther.

[22] Limbert M, Isert D, Klesel N, Markus A, Seeger K, Seibert G, Schrinner E (1991). Antibacterial activities in vitro and in vivo and pharmacokinetics of cefquinome (HR 111 V), a new broad-spectrum cephalosporin. Antimicrob Agents Chemother.

[23] Antonissen G, Devreese M, De Baere S, Hellebuyck T, Van de Maele I, Rouffaer L, Stemkens HJ, De Backer P, Martel A, Croubels S (2015). Comparative pharmacokinetics and allometric scaling of carboplatin in different avian species. PLoS One.

[24] Brogard JM, Comte F, Pinget M (1978). Pharmacokinetics of cephalosporin antibiotics. Antibiot Chemother (1971).

[25] Lorian V (1991). Antibiotics in laboratory medicine.

[26] Stegemann MR, Sherington J, Coati N, Brown SA, Blanchflower S (2006). Pharmacokinetics of cefovecin in cats. J Vet Pharmacol Ther.

[27] Stegemann MR, Sherington J, Blanchflower S (2006). Pharmacokinetics and pharmacodynamics of cefovecin in dogs. J Vet Pharmacol Ther.

[28] Adams HR (2001). Veterinary pharmacology and therapeutics.

[29] Plumb D. Plumb’s veterinary drug handbook. 7th edition. Stockholm: PharmaVet Inc.; 2011.

[30] Toutain P, Ferran A, Bousquet-Melou A. Species Differences in Pharmacokinetics and Pharmacodynamics. Comparative Veterinary Pharmacology. Handb Exp Pharmacol. Springer. 2010;199(199):19–48. 10.1007/978-3-642-10324-7_2 20204582

[31] Ringger NC, Brown MP, Kohlepp SJ, Gronwall RR, Merritt K (1998). Pharmacokinetics of ceftriaxone in neonatal foals. Equine Vet J.

[32] Gardner SY, Aucoin DP (1994). Pharmacokinetics of ceftriaxone in mares. J Vet Pharmacol Ther.

[33] Rule R, Quiroga GH, Rubio M, Buschiazzo HO, Buschiazzo PM (1996). The pharmacokinetics of ceftazidime in lactating and non-lactating cows. Vet Res Commun.

[34] Paradis D, Vallee F, Allard S, Bisson C, Daviau N, Drapeau C, Auger F, LeBel M (1992). Comparative study of pharmacokinetics and serum bactericidal activities of cefpirome, ceftazidime, ceftriaxone, imipenem, and ciprofloxacin. Antimicrob Agents Chemother.

[35] Paulfeuerborn W, Muller HJ, Borner K, Koeppe P, Lode H (1993). Comparative pharmacokinetics and serum bactericidal activities of SCE-2787 and ceftazidime. Antimicrob Agents Chemother.

[36] Collard WT, Cox SR, Lesman SP, Grover GS, Boucher JF, Hallberg JW, Robinson JA, Brown SA (2011). Pharmacokinetics of ceftiofur crystalline-free acid sterile suspension in the equine. J Vet Pharmacol Ther.

[37] Hall TL, Tell LA, Wetzlich SE, Mccormick JD, Fowler LW, Pusterla N (2011). Pharmacokinetics of ceftiofur sodium and ceftiofur crystalline free acid in neonatal foals. J Vet Pharmacol Ther.

[38] Meyer S, Giguère S, Rodriguez R, Zielinski RJ, Grover GS, Brown SA (2009). Pharmacokinetics of intravenous ceftiofur sodium and concentration in body fluids of foals. J Vet Pharmacol Ther.

[39] Amer AM, Fahim EM, Ibrahim RK (1998). Effect of aflatoxicosis on the kinetic behaviour of ceftiofur in chickens. Res Vet Sci.

[40] Tang S, Xiao J, Guo G, He J, Hao Z, Xiao X (2010). Preparation of a newly formulated long-acting ceftiofur hydrochloride suspension and evaluation of its pharmacokinetics in pigs. J Vet Pharmacol Ther.

[41] Brown SA, Chester ST, Robb EJ (1996). Effects of age on the pharmacokinetics of single dose ceftiofur sodium administered intramuscularly or intravenously to cattle. J Vet Pharmacol Ther.

[42] Hwang YH, Song IB, Lee HK, Kim TW, Kim MS, Lim JH, Park BK, Yun HI (2011). Pharmacokinetics and bioavailability of cefquinome in rabbits following intravenous and intramuscular administration. J Vet Pharmacol Ther.

[43] Winther L, Baptiste KE, Friis C (2011). Antimicrobial disposition in pulmonary epithelial lining fluid of horses, part III. cefquinome. J Vet Pharmacol Ther.

[44] Xie W, Zhang X, Wang T, Du S (2013). Pharmacokinetic analysis of cefquinome in healthy chickens. Br Poult Sci.

[45] Li XB, Wu WX, Su D, Wang ZJ, Jiang HY, Shen JZ (2008). Pharmacokinetics and bioavailability of cefquinome in healthy piglets. J Vet Pharmacol Ther.

[46] Guglick MA, MacAllister CG, Clarke CR, Pollet R, Hague C, Clarke JM (1998). Pharmacokinetics of cefepime and comparison with those of ceftiofur in horses. Am J Vet Res.

[47] Pawar YG, Sharma SK (2008). Influence of E. coli lipopolysaccharide induced fever on the plasma kinetics of cefepime in cross-bred calves. Vet Res Commun.

[48] Ismail MM (2005). Disposition kinetics, bioavailability and renal clearance of cefepime in calves. Vet Res Commun.

[49] Gardner SY, Papich MG (2001). Comparison of cefepime pharmacokinetics in neonatal foals and adult dogs. J Vet Pharmacol Ther.

[50] Chiu LM, Menhinick AM, Johnson PW, Amsden GW (2002). Pharmacokinetics of intravenous azithromycin and ceftriaxone when administered alone and concurrently to healthy volunteers. J Antimicrob Chemother.

[51] Bacher K, Schaeffer M, Lode H, Nord CE, Borner K, Koeppe P (1992). Multiple dose pharmacokinetics, safety, and effects on faecal microflora, of cefepime in healthy volunteers. J Antimicrob Chemother.

[52] Barbhaiya RH, Forgue ST, Gleason CR, Knupp CA, Pittman KA, Weidler DJ, Movahhed H, Tenney J, Martin RR (1992). Pharmacokinetics of cefepime after single and multiple intravenous administrations in healthy subjects. Antimicrob Agents Chemother.

[53] Courtin F, Craigmill AL, Wetzlich SE, Gustafson CR, Arndt TS (1997). Pharmacokinetics of ceftiofur and metabolites after single intravenous and intramuscular administration and multiple intramuscular administrations of ceftiofur sodium to dairy goats. J Vet Pharmacol Ther.

[54] Goudah A (2007). Pharmacokinetics of ceftiofur after single intravenous and intramuscular administration in camels (Camelus dromedarius). J Vet Pharmacol Ther.

[55] Dumonceaux G, Isaza R, Koch DE, Hunter RP (2005). Pharmacokinetics and i.m. bioavailability of ceftiofur in Asian elephants (Elephas maximus). J Vet Pharmacol Ther.

[56] Yuan L, Sun J, Wang R, Sun L, Zhu L, Luo X, Fang B, Liu Y (2011). Pharmacokinetics and bioavailability of cefquinome in healthy ducks. Am J Vet Res.

[57] Dinakaran V, Dumka VK, Ranjan B, Balaje R, Sidhu PK (2013). Pharmacokinetics following intravenous administration and pharmacodynamics of cefquinome in buffalo calves. Trop Anim Health Prod.

