# Supplementary material for: Modelling concentrations of antimicrobial drugs: comparative pharmacokinetics of cephalosporin antimicrobials and accuracy of allometric scaling in food-producing and companion animals
Source: BMC Vet Res. 2016 Sep 6;12:185. doi: 10.1186/s12917-016-0817-2 (PMC5011836; doi:10.1186/s12917-016-0817-2)
Supplement: Additional file 1: — Pharmacokinetics of cephalosporins in food producing and companion animal species obtained from literature and presented in a table with literature references. We also provide additional graphs displaying the allometric analysis of cefepime, ceftriaxone, ceftiofur and ceftazidime. (PDF 666 kb) [file 12917_2016_817_MOESM1_ESM.pdf]

Additional file 1

Pharmacokinetics of cephalosporins

This additional file contains a table (table S1) with the data collected from pharmacokinetic studies for different cephalosporins and animal species.

Furthermore the figures from allometric analyses performed for cefepime, ceftriaxone and ceftiofur are provided (figures S1 t/m S3). Figure S4 shows the allometric analysis of ceftazidime excluding human data. A bibliography is included at the end of the document.

Table S1: Pharmacokinetic parameters after intravenous administration of early (1st and 2nd) generation cephalosporins for different species of animals.

	Young /adults	No. of animals	Body weight (kg)	Dosage	Plasma protein binding (%)	Elimination half-life (h)	Calculated elimination half-life (h)	Volume of distribution (L/kg)	Clearance (L/kg/h)	Primary route of elimination	Mechanism of excretion	Reference
**Cefadroxil**												
Horses	Foals	5	Unknown	5 mg/kg i.v.		1.09	0.82	0.374 ± 0.014	0.316 ± 0.010			Duffee[1]
**Cefazolin**												
Dogs	Adults	10	15.2 ± 3.9	25 mg/kg i.v.		1.23 ± 0.243[a]	1.03	0.278 ± 0.063	0.1878 ± 0.0456[b]	renal		Dickson[2]
Cattle	Calves	10	unknown	20 mg/kg i.v.	75 ± 4	0.62[a]	0.33	0.165 (SEM 0.021)	0.3498 ± 0.03792 [b]	renal	glomerular filtration	Soback[3]
Horses	Adults	6	420-550	11 mg/kg i.v.		0.67 [a]	0.39	0.135 ± 0.024^	0.2406 ± 0.0252 [b]	renal		Donecker[4]
	Adults	6	398-564	11 mg/kg i.v.	8.3 ± 1.9	0.63 [a]	0.39	0.188	0.3306 ± 0.072 [b]	renal	mainly tubular secretion (27.3% glomerular filtration)	Sams[5]
**Cefapirin**												
Horses	Adults	6	407-523	20 mg/kg i.v.		0.92 ± 0.066	0.20	0.17 ± 0.013	0.598 ± 0.054[c]			Brown[6]

	Adults	2	417-521	20 mg/kg i.v.		1.13						Juzwiak[7]
**Cefalexin**												
Dogs	Adults	6	20.4-30.0	5 mg/kg i.v.		1.96 ± 0.27	1.51	0.37 ± 0.05	0.17 ± 0.04			Chicoine[8]
	Adults	5	10.0-14.0	20 mg/kg i.v.		1.38 ± 0.37 [a]		0.215 (range 0.139-0.226)^				Carli[9]
Cats	Adults	5	4.95 ± 0.54	10 mg/kg i.v.		1.68 ± 0.20	1.63	0.33 ± 0.03	0.14 ± 0.02			Albarellos[10]
Horses	Adults	6	400-450	10 mg/kg i.v.		1.59 ± 0.13	0.85	0.29 ± 0.02	0.2376 ± 0.046 [b]			Villa[11]
	Adults	6	407-639	10 mg/kg i.v.	22.93 ± 7.94	2.02 ± 0.46	0.85	0.25 ± 0.04	0.204 ± 0.044 [b]	renal	unchanged in urine through glomerular filtration and tubular excretion	Davis[12]
**Cefoxitin**												
Dogs	Adults	4	16.4-26.4	30 mg/kg i.v.		1.30 ± 0.44						Petersen[13]
Cats	Adults	5	5.13 ± 0.52	30 mg/kg i.v.		1.61 ± 0.30	1.59	0.32 ± 0.05	0.14 ± 0.02			Alberellos[14]
Cattle	Calves	9	unknown	20 mg/kg i.v.	42-55	1.12 ± 0.12 [a]	0.75	0.3187 ± 0.0950	0.2928 ± 0.1026 [b]	renal	glomerular filtration, partly tubular secretion	Soback[15]

[a] Recalculated from minutes to hours; [b] Recalculated from ml/kg/min; [c] Recalculated from ml/kg/h
Values are reported as value ± SD unless stated otherwise.

Table S1 (continued): Pharmacokinetic parameters after intravenous administration of modern (3rd and 4th) generation cephalosporins for different species of animals.

	Young /adults	No. of animals	Body weight (kg)	Dosage	Plasma protein binding (%)	Elimination half-life (h)	Calculated elimination half-life (h)	Volume of distribution (L/kg)	Clearance (L/kg/h)	Primary route of elimination	Mechanism of excretion	Reference
**Ceftiofur-Na**												
Cattle	Calves	8	33-53	2.2 mg/kg i.v.		16.1 ± 1.54	13.43	0.345 ± 0.0616	0.0178 ± 0.00325	renal	as active metabolite desfuroylceftiofur through active renal secretion as well as glomerular filtration	Brown[16]
Chickens	Adults	10	Unknown	10 mg/kg i.v.		4.23 ± 0.05	4.22	0.1358 ± 0.0002	0.0223 ± 0.0002			Amer[17]
Horses	Foals	6	41-66	5 mg/kg i.v.		7.78 ± 0.13	6.90	0.741 ± 0.071	0.0744 ± 0.00844[c]			Meyer[18]
	Foals	6	41-66	10 mg/kg i.v.		7.99 ± 0.95	5.76	0.643 ± 0.062	0.0774 ± 0.02102[c]			Meyer[18]
	Foals	6	82-101	5 mg/kg i.v.		8.08 ± 0.69	6.47	0.713 ± 0.104	0.0764 ± 0.0135[c]			Meyer[18]
	Foals	6	82-101	10 mg/kg i.v.		8.03 ± 0.71	5.77	0.710 ± 0.111	0.0853 ± 0.0143[c]			Meyer[18]
	Foals	6	53-68	5 mg/kg i.v.		5.17	5.22	0.44 ± 0.06	0.05838 ± 0.00585[c]			Hal[19]
	Adults	15	unknown	1.0 mg/kg i.v.		21.5 (range 19.2-24.5)	6.51	0.769 (range 0.712-0.826)[e]	0.0818 (range 0.0765-0.0871)[c]			Collard[20]
**Ceftiofur-HCl**												
Pigs	Piglets	5	12.4 ± 0.8	5 mg/kg i.v.		11.01 ± 0.41	7.63	0.11 ± 0.01	0.01 ± 0.00			Tang[21]
**Cefovecin**												
Dogs	Adults	12	11.5-15.3	8 mg/kg i.v.		136 ± 12	111.24	0.122 ± 0.011	0.00076 ± 0.00013[c]	mainly renal, small fraction trough bile	mainly unchanged	Stegeman[22]

Cats	Adults	12	2.9-4.8	8 mg/kg i.v.		184 ± 12	178.2	0.090 ± 0.010	0.00035 ± 0.00004 [c]	renal	mainly unchanged	Stegeman[23]
**Cefoperazone**												
Dogs	Adults	5	10.0-14.0	20 mg/kg i.v.		1.40 ±0.36[a]	1.37	0.233 (range 0.191-0.299)	0.1176 (range 0.0846-0.1488) [b]	bile	Unchanged (?)	Montesiss[24]
Cattle	Calves	5	130	15 mg/kg i.v.		0.89 ± 0.057 [a]				bile		Carli[25]
	Calves	5	130	30 mg/kg i.v.		0.92 ± 0.028 [a]				bile		Carli[25]
	Calves	10	unknown	20 mg/kg i.v.		2.13 ± 0.47 [a]	1.01	0.713 ± 0.167	0.4896 ± 0.096 [b]	in part renal clearance	partly tubular secretion	Soback[26]
Horses	Adults	6	360-470	30 mg/kg i.v.		0.77 ± 0.19	0.65	0.68 ± 0.10	0.72 ± 0.10	extra-renal mechanism		Soraci[27]
Rabbits	Adults	10	1.8 ± 0.1	30 mg/kg i.v.		0.5 ± 0.1	0.32	0.222 ± 0.11[illegible]	0.48 ± 0.17[d]	urinary excretion and biliary excretion		Marino[28]
**Ceftazidime**												
Dogs	Puppies	4	0.8-0.9	20 mg/kg i.v.		0.87	0.79	0.43	0.378 [b]	renal		Kita[29]
	Adults	5	8.0-13.0	20 mg/kg i.v.		0.86	0.76	0.21	0.192 [b]	renal		Kita[29]
	Adults	5	10.0-12.0	20 mg/kg i.v.		1.09 ± 0.03	1.07	0.353 ± 0.0195 [e]	0.228 ± 0.009 [c]	renal		Sakamoto[30]
Cats	Adults	5	5.21 ± 1.45	30 mg/kg i.v.		0.77 ± 0.06	0.66	0.18 ± 0.04	0.19 ± 0.08	renal	glomerular filtration	Albarellos[31]
Cattle	Calves	9	unknown	10 mg/kg i.v.		2.31 ± 0.393 [a]	1.94	0.294 ± 0.064	0.105 ± 0.0156 [b]	renal		Soback[32]
	Adults	5 lactating	450 ± 50	10 mg/kg i.v.		1.1 ± 0.2	4.68	0.4898 ± 0.1369 [e]	0.0725 ± 0.0181 [c]			Rule[33]
	Adults	5 non-lactating	450 ± 50	10 mg/kg i.v.		1.4 ± 0.3	1.45	0.3902 ± 0.2129 [e]	0.1859 ± 0.0442 [c]			Rule[33]

Rabbits	Adults	5	1.9-3.3	20 mg/kg i.v.		0.73 ± 0.05	0.73	0.289 ± 0.017 [e]	0.276 ± 0.0108 [c]	renal		Sakamoto[30]
Humans	Adults	15	72.0	13.89 mg/kg i.v.		1.95 ± 0.25	1.54	0.21 ± 0.03	0.0948 ± 0.014[a]			Paradis[34]
Humans	Adults	12	70.8	28.57 mg/kg i.v.		1.65 ± 0.22	2.05	0.204 ± 0.02	0.069 ± 0.011[f]			Paulfeuerborn[35]
**Ceftriaxone**												
Dogs	Adults	6	19.6 ± 2.2	50 mg/kg i.v.		0.88	0.89	0.277 ± 0.07	0.2166 ± 0.0468 [b]			Rebuelto[36]
Cats	Adults	5	5.51 ± 1.83	25 mg/kg i.v.		1.73 ± 0.23	1.07	0.57 ± 0.22	0.37 ± 0.13	renal	glomerular filtration and tubular secretion and/or nonrenal excretion (bile)	Albarellos[37]
Cattle	Adults	8	unknown	6.72 mg/kg i.v.		1.02 ± 0.07	1.16	0.50 ±0.19	0.30 ± 0.09			Kumar[38]
Horses	Foals	5	60-79.5	25 mg/kg i.v.		3.25 ± 3.22	1.93	1.605 ± 1.083 [e]	0.575 ± 0.3192 [c]			Ringger[39]
	Adults	6	453 (361-541)	14 mg/kg i.v.		0.81 ± 0.16	0.62	0.15 ± 0.02	0.1686 ± 0.0324 [b]			Gardner[40]
**Cefotaxime**												
Dogs	Adults	4	13.87 ± 1.19	50 mg/kg i.v.		0.74 ± 0.12	0.53	0.48 ± 0.23	0.63 ± 0.17			Guerrini[41]
Cats	Adults	6	unknown	10 mg/kg i.v.		0.977 ± 0.254	0.76	0.182 ± 0.034	0.1655 ± 0.07494 [b]			McElroy[42]
Cattle	Calves	5	100	10 mg/kg i.v.		3.48 ± 1.17	3.00	3.51 ± 0.66	0.81 ± 0.10		low recovery in urine	Sharma[43]
Horses	Foals	5	24 (16-43)	40 mg/kg i.v.		0.60	0.65	0.29 ± 0.07	0.31 ± 0.03		active metabolite desacetyl-cefotaxime	Gardner[44]
	Adults	5	588.8 ± 18.08	25 mg/kg i.v.		0.97 (SEM 0.13) [a]	0.34	0.214 (SEM 0.0134) [e]	0.432 (SEM 0.023) [b]			Orsini[45]
**Cefquinome**												

Dogs	Adults	3	21.8 ± 0.8	5 mg/kg i.v.	<10	0.85 ± 0.10	0.72	0.20 ± 0.06	0.1929 ± 0.037[d]	renal		Limbert[46]
	Adults	3	21.5 ± 2.2	10 mg/kg i.v.	<10	0.98 ± 0.28	0.75	0.24 ± 0.09	0.2213 ± 0.078[d]	renal		Limbert[46]
	Adults	3	21.2 ± 2.6	20 mg/kg i.v.	<10	0.96 ± 0.08	0.80	0.22 ± 0.04	0.1913 ± 0.015[d]	renal		Limbert[46]
Cattle	Calves	4	118.5 ± 8.9	10 mg/kg i.v.	<10	1.33 ± 0.41	1.21	0.23 ± 0.13	0.1322 ± 0.065[d]	renal		Limbert[46]
Pigs	Piglets	5	20-25	2 mg/kg i.v.		1.85 ± 1.11	1.23	0.46	0.26			Li[47]
	Adults	5	18.2 ± 0.5	10 mg/kg i.v.	<10	1.32 ± 0.18	1.04	0.24 ± 0.11	0.1599 ± 0.055[d]	renal		Limbert[46]
Chickens	Adults	10	1.8-2.2	2 mg/kg i.v.		1.29 ± 0.10	0.97	0.49 ± 0.05	0.35 ± 0.04			Xie[48]
Horses	Adults	6	579 ± 57	1 mg/kg i.v.	20.8-30.2	2.77	1.21	0.21	0.12			Winther[49]
Rabbits	Adults	12	2.8-3.2	2 mg/kg i.v.		0.93 ± 0.14	0.81	0.21 ± 0.03	0.18 ± 0.05			Hwang[50]
**Cefepime**												
Dogs	Adults	6	7.25-11.25	14 mg/kg i.v.		1.09 ± 0.27	0.75	0.14 ± 0.04	0.13 ± 0.04	renal		Gardner[51]
Cattle	Calves	4	156.8	6.5 mg/kg i.v.		2.38 ± 0.16	2.21	0.21 ± 0.01	0.066 ± 0.0048[b]	renal	glomerular filtration	Ismail[52]
	Calves	4	92-100	10 mg/kg i.v. (healthy)		1.26 ± 0.01	1.66	0.25 ± 0.01	0.1044 ± 0.027[b]	renal	unchanged	Pawer[53]
	Calves	4	92-100	10 mg/kg i.v. (febrile)		1.62 ± 0.09	1.94	0.32 ±0.01	0.1142 ± 0.012[b]	renal	unchanged	Pawer[53]
Horses	Foals	5	50-66	14 mg/kg i.v.		1.65 ± 0.10	1.56	0.18 ± 0.05	0.08 ± 0.02	renal		Gardner[51]
	Adults	6	410-573	2.2 mg/kg i.v.		2.095 ± 1.253[a]	2.20	0.225 ± 0.077[e]	0.0708 ± 0.0108[b]			Guglick[54]
**Cefpirome**												
Dogs	Adults	5	10.0-12.0	20 mg/kg i.v.		1.20 ± 0.11	1.22	0.371 ± 0.0476[e]	0.210 ± 0.0108[b]	renal		Sakamoto[30]
	Adults	5	8.0-13.0	20 mg/kg i.v.		0.90	0.79	0.22	0.192[b]			Kita[29]

	Adults	5	18.5-24.5	10 mg/kg i.v.		1.05 [a]						Klesel[55]
Rabbits	Adults	5	1.9-3.3	20 mg/kg i.v.		0.79 ± 0.05	0.78	0.269 ± 0.0173 [e]	0.240 ± 0.0102 [b]	renal		Sakamoto[30]
	Adults	4	2.3	20 mg/kg i.v.		1.48 [a]				renal		Klesel [55]

[a] Recalculated from minutes to hours; [b] Recalculated from ml/kg/min; [c] Recalculated from ml/kg/h; [d] Recalculated from total clearance in ml/min; [e] Recalculated from ml/kg; [f] Recalculated from ml/min/1.73 m^2 (assuming 1.73 m^2 equals a 70 kg weighing human)
Values are reported as value ± SD unless stated otherwise.

Figure S1: Allometric analysis performed for cefepime.

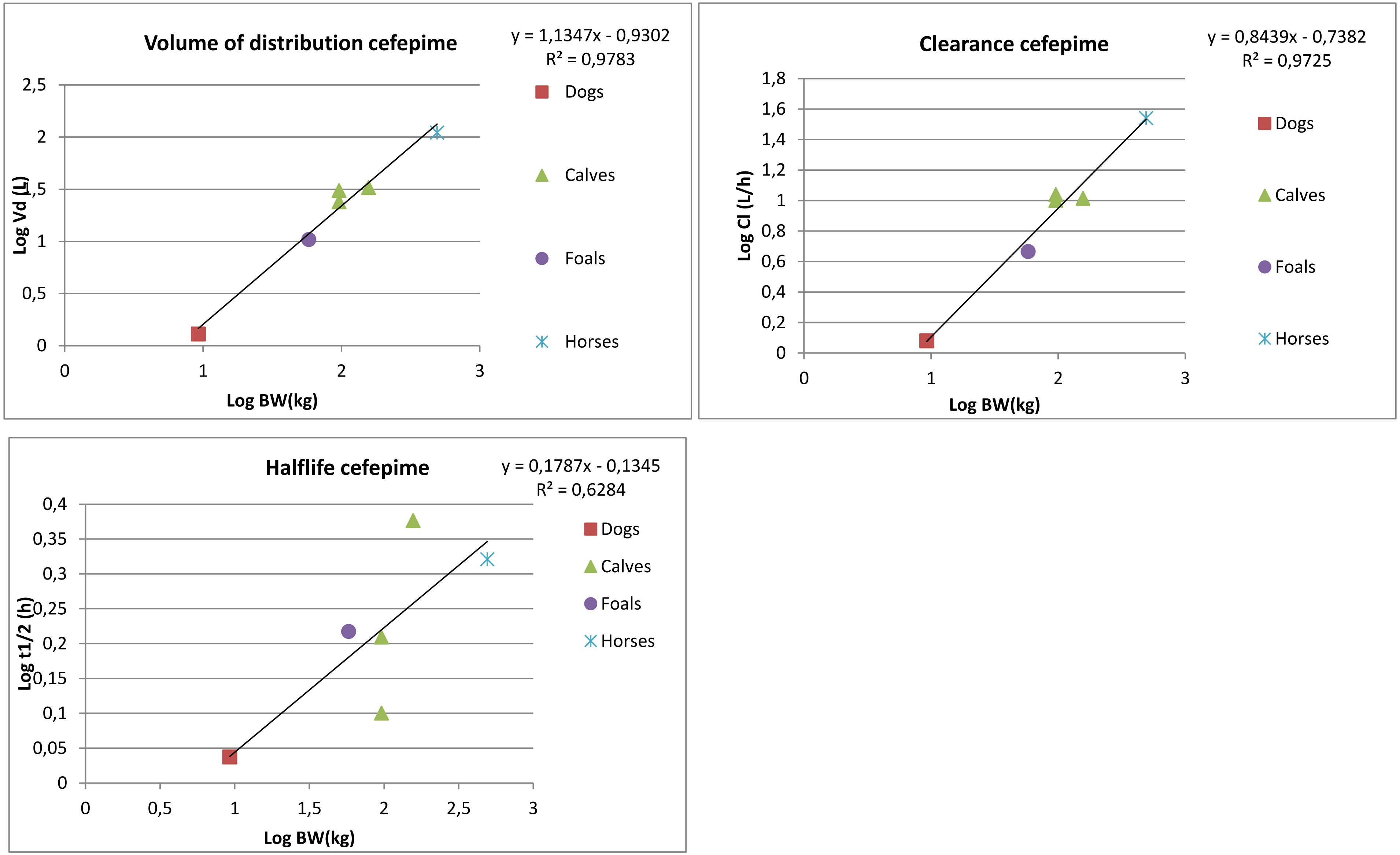

Figure S2: Allometric analysis performed for ceftriaxone

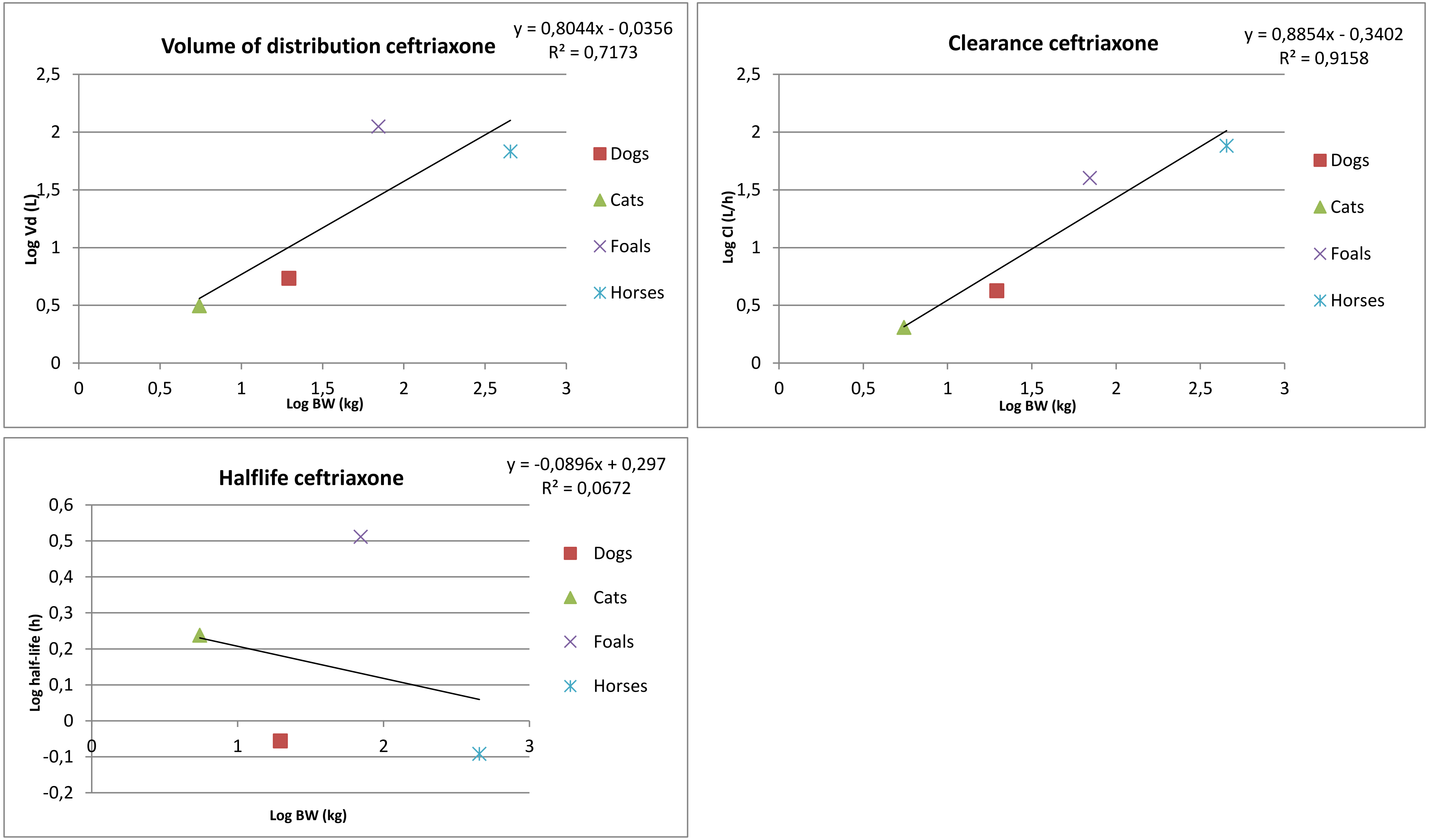

Figure S3: Allometric analysis performed for ceftiofur

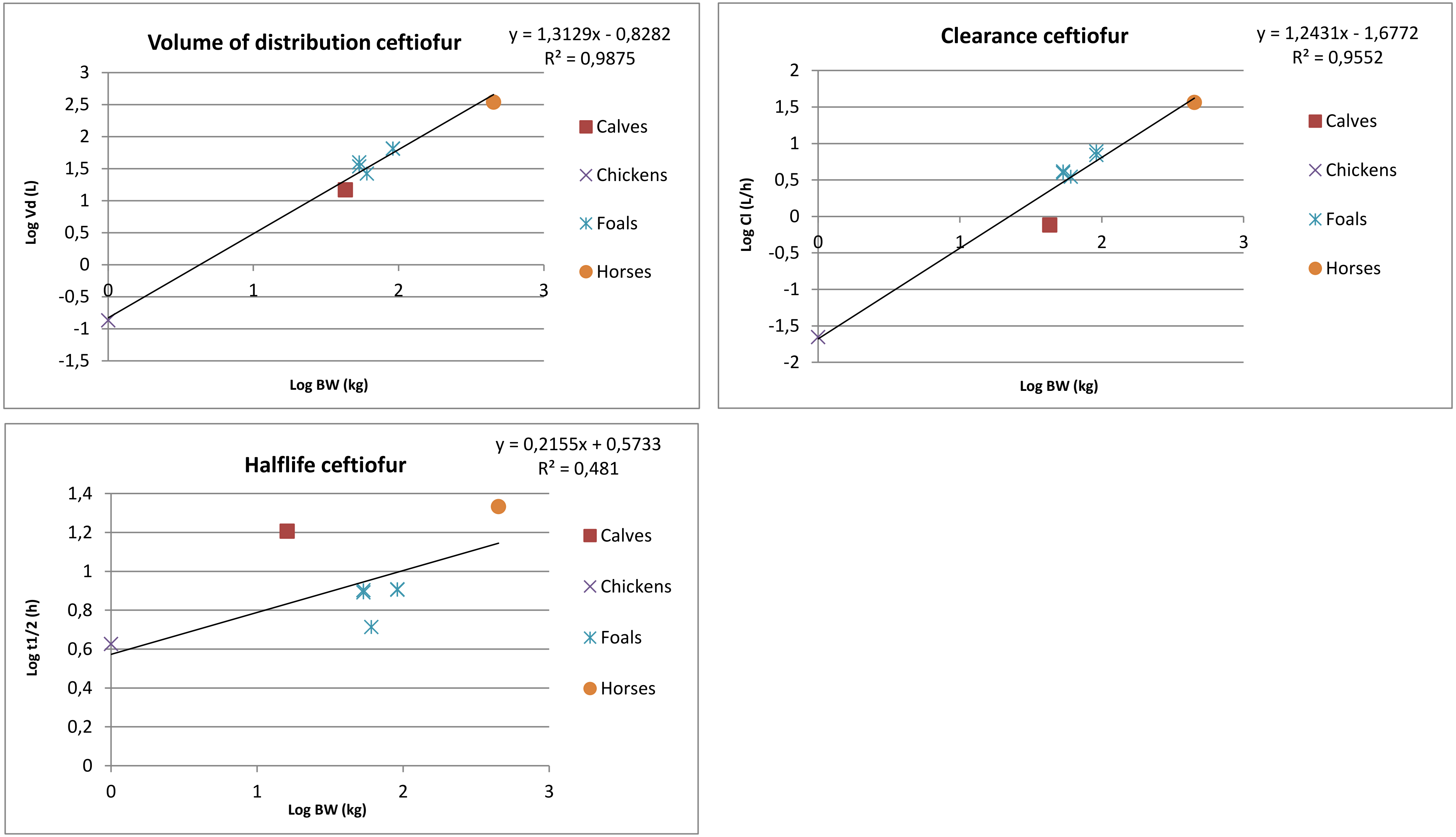

Figure S4: Allometric analysis performed for ceftazidime excl. human data.

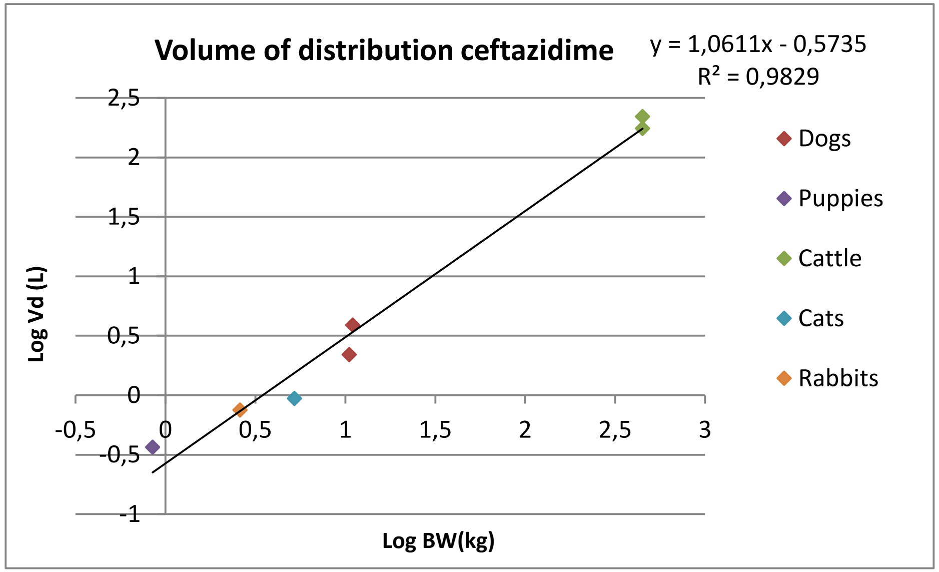

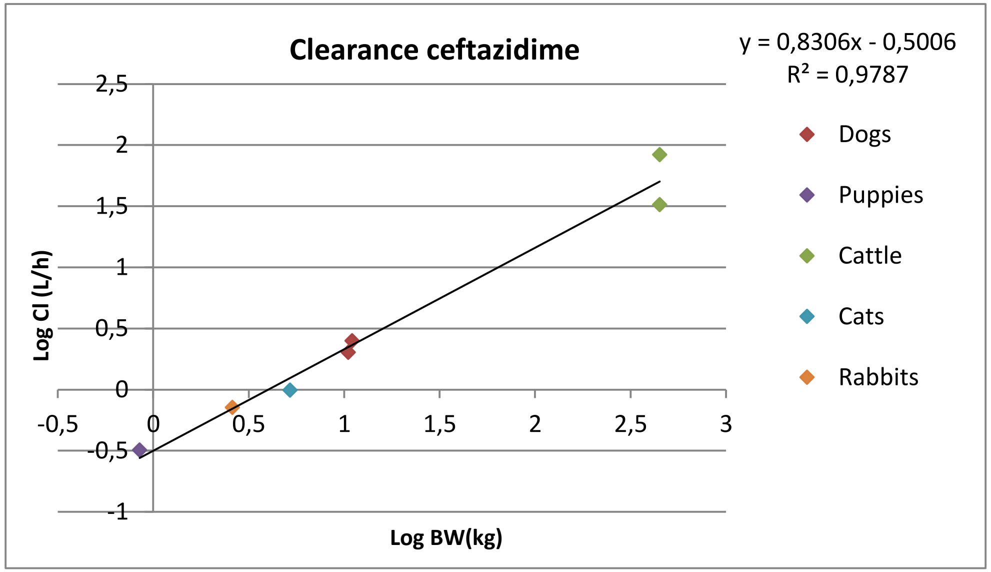

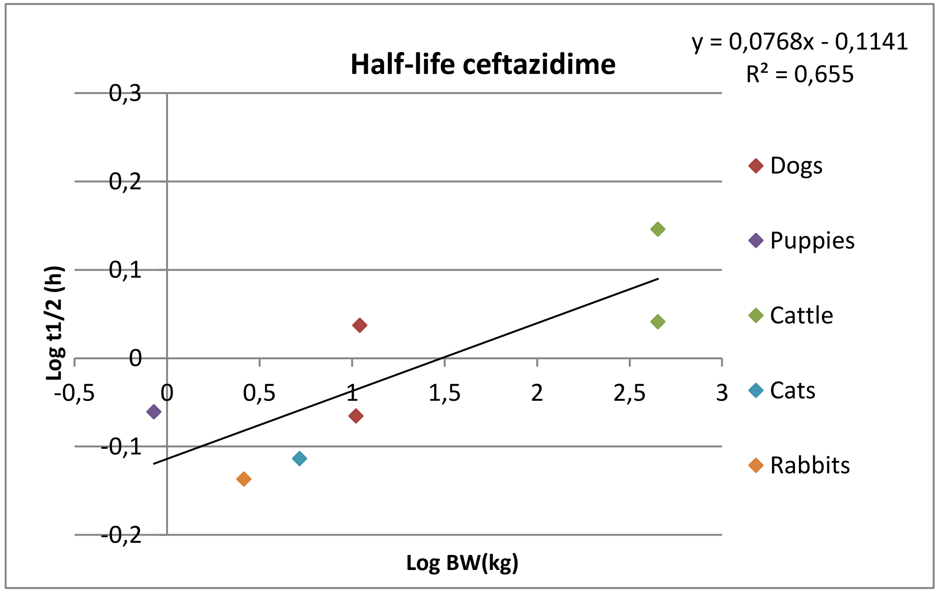

References

1. Duffee NE, Stang BE, Schaeffer DJ (1997) The pharmacokinetics of cefadroxil over a range of oral doses and animal ages in the foal. J Vet Pharmacol Ther 20(6):427-433
2. Dickson PL, DiPiro JT, Michael KA, Cheung RP, Hall EM (1987) Effect of hemorrhagic shock on cefazolin and gentamicin pharmacokinetics in dogs. Antimicrob Agents Chemother 31(3):389-392
3. Soback S, Ziv G, Kurtz B, Paz R (1987) Clinical pharmacokinetics of five oral cephalosporins in calves. Res Vet Sci 43(2):166-172
4. Donecker JM, Sams RA, Ashcraft SM (1986) Pharmacokinetics of probenecid and the effect of oral probenecid administration on the pharmacokinetics of cefazolin in mares. Am J Vet Res 47(1):89-95
5. Sams RA, Ruoff WW,Jr (1985) Pharmacokinetics and bioavailability of cefazolin in horses. Am J Vet Res 46(2):348-352
6. Brown MP, Gronwall RR, Houston AE (1986) Pharmacokinetics and body fluid and endometrial concentrations of cephapirin in mares. Am J Vet Res 47(4):784-788
7. Juzwiak JS, Brown MP, Gronwall R, Houston AE (1989) Effect of probenecid administration on cephapirin pharmacokinetics and concentrations in mares. Am J Vet Res 50(10):1742-1747
8. Chicoine A, Cox W, Huang L, Wang G, Dowling P (2009) Bioavailability and pharmacokinetics of a novel cephalexin oral paste formulation in fed and fasted dogs. J Vet Pharmacol Ther 32(4):400-402
9. Carli S, Anfossi P, Villa R, Castellani G, Mengozzi G, Montesissa C (1999) Absorption kinetics and bioavailability of cephalexin in the dog after oral and intramuscular administration. J Vet Pharmacol Ther 22(5):308-313
10. Albarellos GA, Montoya L, Quaine PC, Landoni MF (2011) Pharmacokinetics and bioavailability of a long-acting formulation of cephalexin after intramuscular administration to cats. Res Vet Sci 91(1):129-131
11. Villa R, Belloli C, Cagnardi P, Sonzogni O, Bacchetta S, Carli S (2002) Pharmacokinetics of cephalexin in the horse after intravenous and intramuscular administration of two formulations. Veterinary Journal 164(1):74-76
12. Davis JL, Salmon JH, Papich MG (2005) Pharmacokinetics and tissue fluid distribution of cephalexin in the horse after oral and i.v. administration. J Vet Pharmacol Ther 28(5):425-431
13. Petersen SW, Rosin E (1995) Cephalothin and cefazolin in vitro antibacterial activity and pharmacokinetics in dogs. Vet Surg 24(4):347-351
14. Albarellos GA, Montoya L, Quaine PC, Lupi MP, Landoni MF (2010) Pharmacokinetics of cefoxitin after intravenous and intramuscular administration to cats. J Vet Pharmacol Ther 33(6):619-621
15. Soback S (1988) Pharmacokinetics of single doses of cefoxitin given by the intravenous and intramuscular routes to unweaned calves. J Vet Pharmacol Ther 11(2):155-162
16. Brown SA, Chester ST, Robb EJ (1996) Effects of age on the pharmacokinetics of single dose ceftiofur sodium administered intramuscularly or intravenously to cattle. J Vet Pharmacol Ther 19(1):32-38
17. Amer AM, Fahim EM, Ibrahim RK (1998) Effect of aflatoxicosis on the kinetic behaviour of ceftiofur in chickens. Res Vet Sci 65(2):115-118
18. Meyer S, Giguère S, Rodriguez R, Zielinski RJ, Grover GS, Brown SA (2009) Pharmacokinetics of intravenous ceftiofur sodium and concentration in body fluids of foals. J Vet Pharmacol Ther 32(4):309-316
19. Hall TL, Tell LA, Wetzlich SE, Mccormick JD, Fowler LW, Pusterla N (2011) Pharmacokinetics of ceftiofur sodium and ceftiofur crystalline free acid in neonatal foals. J Vet Pharmacol Ther 34(4):403-409
20. Collard WT, Cox SR, Lesman SP, Grover GS, Boucher JF, Hallberg JW, Robinson JA, Brown SA (2011) Pharmacokinetics of ceftiofur crystalline-free acid sterile suspension in the equine. J Vet Pharmacol Ther 34(5):476-481
21. Tang S, Xiao J, Guo G, He J, Hao Z, Xiao X (2010) Preparation of a newly formulated long-acting ceftiofur hydrochloride suspension and evaluation of its pharmacokinetics in pigs. J Vet Pharmacol Ther 33(3):238-245
22. Stegemann MR, Sherington J, Blanchflower S (2006) Pharmacokinetics and pharmacodynamics of cefovecin in dogs. J Vet Pharmacol Ther 29(6):501-511
23. Stegemann MR, Sherington J, Coati N, Brown SA, Blanchflower S (2006) Pharmacokinetics of cefovecin in cats. J Vet Pharmacol Ther 29(6):513-524
24. Montesissa C, Villa R, Anfossi P, Zanoni R, Carli S (2003) Pharmacodynamics and pharmacokinetics of cefoperazone and cefamandole in dogs following single dose intravenous and intramuscular administration. Veterinary Journal 166(2):170-176
25. Carli S, Montesissa C, Sonzogni O, Madonna M (1986) Pharmacokinetic of sodium cefoperazone in calves. Pharmacol Res Commun 18(5):481-490
26. Soback S, Ziv G (1989) Pharmacokinetics of single doses of cefoperazone given by the intravenous and intramuscular routes to unweaned calves. Res Vet Sci 47(2):158-163

27. Soraci AL, Mestorino ON, Errecalde JO (1996) Pharmacokinetics of cefoperazone in horses. J Vet Pharmacol Ther 19(1):39-43
28. Marino EL, Fernandez Lastra C, Gonzalez Alonso I, Dominguez-Gil A (1987) Disposition and excretion of cefoperazone in rabbits. Arzneimittelforschung 37(3):345-349
29. Kita Y, Yamazaki T, Imada A (1992) Comparative pharmacokinetics of SCE-2787 and related antibiotics in experimental animals. Antimicrob Agents Chemother 36(11):2481-2486
30. Sakamoto H, Hatano K, Higashi Y, Mine Y, Nakamoto S, Tawara S, Kamimura T, Matsumoto F, Kuwahara S (1993) Animal pharmacokinetics of FK037, a novel parenteral broad-spectrum cephalosporin. J Antibiot (Tokyo) 46(1):120-130
31. Albarellos GA, Ambros LA, Landoni MF (2008) Pharmacokinetics of ceftazidime after intravenous and intramuscular administration to domestic cats. Vet J 178(2):238-243
32. Soback S, Ziv G (1989) Pharmacokinetics of ceftazidime given alone and combination with probenecid to unweaned calves. Am J Vet Res 50(9):1566-1569
33. Rule R, Quiroga GH, Rubio M, Buschiazzo HO, Buschiazzo PM (1996) The pharmacokinetics of ceftazidime in lactating and non-lactating cows. Vet Res Commun 20(6):543-550
34. Paradis D, Vallee F, Allard S, Bisson C, Daviau N, Drapeau C, Auger F, LeBel M (1992) Comparative study of pharmacokinetics and serum bactericidal activities of cefpirome, ceftazidime, ceftriaxone, imipenem, and ciprofloxacin. Antimicrob Agents Chemother 36(10):2085-2092
35. Paulfeuerborn W, Muller HJ, Borner K, Koeppe P, Lode H (1993) Comparative pharmacokinetics and serum bactericidal activities of SCE-2787 and ceftazidime. Antimicrob Agents Chemother 37(9):1835-1841
36. Rebuelto M, Albarellos G, Ambros L, Kreil V, Montoya L, Bonafine R, Otero P, Hallu R (2002) Pharmacokinetics of ceftriaxone administered by the intravenous, intramuscular or subcutaneous routes to dogs. J Vet Pharmacol Ther 25(1):73-76
37. Albarellos GA, Kreil VE, Landoni MF (2007) Pharmacokinetics of ceftriaxone after intravenous, intramuscular and subcutaneous administration to domestic cats. J Vet Pharmacol Ther 30(4):345-352
38. Kumar S, Srivastava AK, Dumka VK, Kumar N, Raina RK (2010) Plasma pharmacokinetics and milk levels of ceftriaxone following single intravenous administration in healthy and endometritic cows. Vet Res Commun 34(6):503-510
39. Ringger NC, Brown MP, Kohlepp SJ, Gronwall RR, Merritt K (1998) Pharmacokinetics of ceftriaxone in neonatal foals. Equine Vet J 30(2):163-165
40. Gardner SY, Aucoin DP (1994) Pharmacokinetics of ceftriaxone in mares. J Vet Pharmacol Ther 17(2):155-156
41. Guerrini VH, English PB, Filippich LJ, Schneider J, Bourne DW (1986) Pharmacokinetics of cefotaxime in the dog. Vet Rec 119(4):81-83
42. McElroy D, Ravis WR, Clark CH (1986) Pharmacokinetics of cefotaxime in the domestic cat. Am J Vet Res 47(1):86-88
43. Sharma SK, Srivastava AK, Bal MS (1995) Disposition kinetics and dosage regimen of cefotaxime in cross-bred male calves. Vet Res 26(3):168-173
44. Gardner SY, Sweeney RW, Divers TJ (1993) Pharmacokinetics of cefotaxime in neonatal pony foals. Am J Vet Res 54(4):576-579
45. Orsini JA, Moate PJ, Engiles J, Norman T, Poppenga R, Benson CE, Boston RC (2004) Cefotaxime kinetics in plasma and synovial fluid following intravenous administration in horses. J Vet Pharmacol Ther 27(5):293-298
46. Limbert M, Isert D, Klesel N, Markus A, Seeger K, Seibert G, Schrinner E (1991) Antibacterial activities in vitro and in vivo and pharmacokinetics of cefquinome (HR 111V), a new broad-spectrum cephalosporin. Antimicrob Agents Chemother 35(1):14-19
47. Li XB, Wu WX, Su D, Wang ZJ, Jiang HY, Shen JZ (2008) Pharmacokinetics and bioavailability of cefquinome in healthy piglets. J Vet Pharmacol Ther 31(6):523-527
48. Xie W, Zhang X, Wang T, Du S (2013) Pharmacokinetic analysis of cefquinome in healthy chickens. Br Poult Sci 54(1):81-86
49. Winther L, Baptiste KE, Friis C (2011) Antimicrobial disposition in pulmonary epithelial lining fluid of horses, part III. cefquinome. J Vet Pharmacol Ther 34(5):482-486
50. Hwang YH, Song IB, Lee HK, Kim TW, Kim MS, Lim JH, Park BK, Yun HI (2011) Pharmacokinetics and bioavailability of cefquinome in rabbits following intravenous and intramuscular administration. J Vet Pharmacol Ther 34(6):618-620
51. Gardner SY, Papich MG (2001) Comparison of cefepime pharmacokinetics in neonatal foals and adult dogs. J Vet Pharmacol Ther 24(3):187-192

52. Ismail MM (2005) Disposition kinetics, bioavailability and renal clearance of cefepime in calves. Vet Res Commun 29(1):69-79
53. Pawar YG, Sharma SK (2008) Influence of E. coli lipopolysaccharide induced fever on the plasma kinetics of cefepime in cross-bred calves. Vet Res Commun 32(2):123-130
54. Guglick MA, MacAllister CG, Clarke CR, Pollet R, Hague C, Clarke JM (1998) Pharmacokinetics of cefepime and comparison with those of ceftiofur in horses. Am J Vet Res 59(4):458-463
55. Klesel N, Seeger K (1983) Pharmacokinetic properties of the new cephalosporin antibiotic HR 810 in animals. Infection 11(6):318-321
